# Development and evaluation of an e-learning course in oxygen therapy

**DOI:** 10.1186/s12909-022-03838-1

**Published:** 2022-11-10

**Authors:** Maryam Arabani Nezhad, Haleh Ayatollahi, Hazhir Heidari Beigvand

**Affiliations:** 1 Present Address: Department of Health Information Management, School of Health Management and Information Sciences, Iran University of Medical Sciences, Tehran, Iran; 2 Health Management and Economics Research Center, Health Management Research Institute, Iran University of Medical Sciences, Tehran, Iran; 3 Family Medicine and Public Health Research Center, Faculty of Medicine, Iran University of Medical Sciences, Tehran, Iran

**Keywords:** Distance education, Distance learning, Online learning, Oxygen inhalation therapy, Respiratory tract diseases, Medical informatics

## Abstract

**Background:**

Respiratory problems are among the most challenging situations in emergency care services. Different oxygen therapy methods are usually used to deal with these problems. In recent years, oxygen therapy has been recognized as one of the most widely used therapeutic processes in emergency departments (ED) mainly due to the Covid-19 pandemic. The aim of this study was to develop and evaluate an e-learning course in oxygen therapy for the ED clinicians.

**Methods:**

This was a pre-post study conducted in three phases in 2021. Initially, the educational requirements of clinicians (*n* = 181) were investigated using a questionnaire, and in the second phase, an interactive e-learning course was developed. In the third phase, the course was assessed in terms of maintaining the principles of developing an e-learning course, affecting participants’ knowledge, and supporting usability requirements.

**Results:**

The findings revealed that training in oxygen therapy was essential for the ED clinicians. Therefore, an e-learning course was developed. The content production experts and the participants evaluated the content and usability of the online course at a good level. In addition, there was a statistically significant difference between the nurses’ (*p* < 0.001) and general practitioners’ (*p* < 0.002) pre- and post-test scores suggesting that the course improved their knowledge.

**Conclusion:**

It seems that the e-learning course developed in the current study can improve health care professionals’ knowledge and quality of care. However, more evaluation studies are needed to investigate the effectiveness of the course for other clinicians, such as nurses who work in intensive care units.

## Introduction

Respiratory disorders are among the most common reasons for patient referral to medical centers and emergency departments [1]. Moreover, respiratory diseases are among the most important causes of death in the world [2, 3]. Most of these diseases lead to permanent lung damages and respiratory disorders. Respiratory problems can lengthen the hospital stay, and result in receiving more care. Importantly, early detection of respiratory diseases may reduce the severity of the disease and its complications. Oxygen therapy is one of the methods that are used for improving patient condition or treating patients with respiratory diseases [1, 3]. In particular, with respect to the Covid-19 pandemic and patients’ needs to receive oxygen therapy services, this procedure is known as one of the most common and important services for patients [4, 5].

It is also worth mentioning that in some diseases such as stroke, heart attack, gastrointestinal bleeding, seizures, respiratory disorders, anesthesia, head trauma, and loss of blood and body fluids, the tissues of the body do not receive adequate oxygen and this may cause dangerous consequences such as hypoxemia, bruising, and respiratory failure [6–8]. On the other hand, improper use of oxygen can lead to irreparable damages to patients, and in some cases may cause death in addition to wasting valuable oxygen resources [2, 9, 10]. Pulmonary oxygen poisoning, atelectasis, Pickwickian syndrome, visual disturbances, retinal injuries, and blindness are other side effects of oxygen toxicity [11, 12].

As oxygen therapy is one of the most challenging and widely used therapy in the emergency departments (ED) [5, 10–12], improving ED clinicians’ knowledge and professional skills can play an important role in improving patients’ health condition and preventing problems caused by improper use of oxygen [6, 13, 14]. Moreover, according to the literature, the ED clinicians need to be trained in different subjects, such as pulmonary problems, cardiopulmonary resuscitation, vascular resuscitation, triage, respiratory resuscitation, cardiovascular physical examination, oxygen therapy, airway opening, and respiratory devices [4, 9, 10].. Some studies show that ED nurses and physicians do not have adequate knowledge about the applications and methods of oxygen therapy, which, in turn, may lead to medical errors, patient dissatisfaction, and problems in the treatment process [6, 15, 16].

It is notable that although emergency department (ED) is one of the most important department responsible for treating patients in acute conditions [13], challenges such as the large number of emergency department visits, a variety of treatment methods, and time constraints to provide the best services may increase the risk of medical errors [17–19]. As a result, identifying the most common medical procedures and the educational requirements of the ED staff can play an important role in improving patient care, decreasing medical errors, and reducing the hospital stay time [3, 14, 20]. Some studies have also highlighted the low quality of in-service training for physicians and nurses, especially in the emergency departments and in the clinical fields [14, 21]. Therefore, new learning methods, such as e-learning courses have been adopted to update and improve clinicians’ knowledge and professional skills [22–26]. E-learning includes different components, such as learning activities, access to resources, and learner evaluation via the Internet and people can get access to these courses using their own computers or mobile phones [27, 28]. This method enables all learners to get access to the course contents at any time and place using the Internet and other information and communication technologies (ICT) [27, 29].

So far, a number of online courses, such as online training module for identifying and treating human trafficking victims [30], managing circulatory shock [31], identifying and managing alcohol withdrawal [32], interpreting electrocardiograms (ECGs) [33], and using manual defibrillators in cardiopulmonary resuscitation [34] have been developed for ED staff. However, few courses are related to the field of oxygen therapy. The aim of these courses was mainly developing a blended course for airway management in the emergency department [14], setting up an e-learning resource for the emergency department nurses in the field of oxygen therapy services [35], and providing e-learning modules for airway management in intensive care unit (ICU) [6]. It is notable that in these courses, mainly blended learning methods were applied, and they were not fully online. Given the importance of quick decision making in the emergency department and the role of oxygen therapy as an effective process in improving or treating patient condition, especially in patients with Covid-19 [35, 36], the aim of this study was developing an online e-learning course for oxygen therapy which included both concepts and methods. Such an online course could help the participants to learn what they needed at any time and place without the need for attending in-person classes.

## Methods

This was a before-after (pre-post) study which was conducted in three phases in 2021. Prior to the research, the code of ethics (IR.IUMS.REC.1397.603) was received from the National Ethics Committee in Biomedical Research.

### Phase 1: educational needs assessment

In the first phase, the educational needs of ED clinicians were investigated to develop an oxygen therapy course. Initially, the reference books, such as Rosen’s emergency medicine: concepts and clinical practice [35], Tintinalli’s emergency medicine: A comprehensive study guide [37], and Roberts and Hedges’ clinical procedures in emergency medicine and acute Care [3] were used, and 27 topics related to the oxygen therapy concepts and methods were extracted. These topics were related to the main concepts and definitions (16 items) as well as different methods (11 items) of oxygen therapy. Then, a questionnaire with two options “necessary” and “unnecessary” was designed to include these items and administered to the physicians and nurses who worked in five different emergency departments. The potential participants were 182 nurses, 36 general practitioners, and 25 emergency physicians.

The face and content validity of the questionnaire were assessed by six emergency physicians. They reviewed the clarity of the questions, and agreed that the questionnaire adequately contained relevant and appropriate items related to the oxygen therapy concepts and methods. The content validity was also determined by using Lawshe’s index [38]. The emergency physicians were requested to specify which items were necessary to be included in the questionnaire. The scale composed of three options, namely, not necessary, useful but not essential, and essential. Then, content validity ratio (CVR) was computed for each item. As the acceptable value for CVR was 0.99 based on the Lawshes’s table [38], the items with values less than 0.99 were removed from the questionnaire. These items included 4 topics from the main concepts and definitions: physical evaluation of the respiratory system, invasive oxygen therapy, demand-based oxygen delivery system, and hyperbaric oxygen therapy, and 2 topics from different methods of oxygen therapy which were nasopharyngeal catheter and tracheostomy necklace. The CVR and content validity index (CVI) was 1 for the remaining items in the questionnaire. The reliability of the questionnaire was calculated using KR-20 correlation coefficient (KR-20 = 0.91). In order to analyze data, SPSS software version 21 and descriptive statistics (frequency, mean value, and standard deviation) were used.

### Phase 2: developing an interactive e-learning course

In the second phase, the required contents were extracted from the mentioned textbooks and reference books [3, 35, 37], and guidelines approved by national and international organizations, such as the World Health Organization (WHO) [39], National Institutes of Health (NIH) [40], and the Ministry of Health and Medical Education of Iran [41]. Then, the course was developed using interactive content creation software, such as Camtasia, Flip PDF, and Storyline. The contents of the course were voiced, and pre- and post-test questions were defined under the supervision of an emergency physician (HHB) and uploaded to the website: www.oxygentherapy.ir.

### Phase 3: e-learning course evaluation

The e-learning course was evaluated using three different methods. Initially, the course was presented to 15 content production experts, who had at least 2 years of work experience in developing, producing, and evaluating e-learning courses and were employed in three different medical universities. They were asked to complete a 5-point Likert scale questionnaire in which the participants’ responses ranged from very low (1) to very high (5). This questionnaire consisted of four sections including cultural and literary criteria (3 questions); educational elements including objectives, content, methods, evaluation, and resources (24 questions); course guide (3 questions); and artistic and technological criteria (10 questions). It was designed based on “the guidelines for continuing virtual education programs of the medical community” provided by the Ministry of Health of Iran [42].

Then, all physicians (7 emergency physicians and 5 general practitioners) and 49 nurses, who worked in an emergency department, were invited to participate in the course and respond to the pre-test and post-test questions. The tests consisted of 20 multiple choice questions designed under the supervision of an emergency physician (HHB). This course was available for 2 months and the completion of the course by the participants was checked by one the researchers (MA) through the site management panel. Moreover, reminder messages were sent to the clinicians to complete the course. To analyze the data, the Kolmogorov-Smirnov test was used for each group of the participants, and paired t-test was used to examine the significance of the difference between the means in each group.

Finally, at the end of the course, the participants were asked to complete a 10-point Likert scale usability evaluation questionnaire (QUIS, 5.5). The questionnaire included 31 items in six sections, participant’s personal information (4 items), overall reaction to the course (6 items), screen design (4 items), terminology and course information (6 items), learnability (6 items), and general features (5 items). The validity of the questionnaire has already been assessed in other studies and the reliability of the questionnaire was calculated (α = 0.93). To analyze the data, descriptive statistics including mean value and standard deviation were used. We also calculated the mean value for each part of the questionnaire and reported it in three levels of zero to 3 (weak), 3.1 to 6 (average), and 6.1 to 9 (good).

## Results

The results of the current study are presented in three following sections.

### Topics for the e-learning course

In the first phase of the study, 138 nurses (74%), 28 general practitioners (70%), and 15 emergency physicians (60%) completed the “educational needs assessment” questionnaire. Among nurses, most of the participants were female (*n* = 111, 80.4%) and the age range of 30–41 (*n* = 61, 44.3%) had the highest frequency. More than half of the general practitioners were male (*n* = 16, 57.1%) and the highest frequency belonged to the age range of 41–50 (*n* = 15, 53.6%). Similarly, most of the emergency physicians were male (*n* = 10, 66.6%) and the age group of 51–60 had the highest frequency (*n* = 9, 60%).

The mean age of the nurses participated in the study was 35 ± 2.42 and the mean age of the physicians was 45 ± 2.12. Table 1 shows the participants’ demographic characteristics in the first phase of the research.

**Table 1:** Participants’ characteristics in the first phase of the research

Participants		Nurses	General practitioners	Emergency physicians
Individual variables		Fr (%)	Fr (%)	Fr (%)
Sex	Male	27 (19.6)	16 (57.1)	10 (66.6)
	Female	111 (80.4)	12 (42.9)	5 (33.4)
Age	20–30	44 (31.8)	4 (14.3)	0
	31–40	61 (44.3)	9 (32.1)	0
	41–50	33 (23.9)	15 (53.6)	6 (40)
	51–60	0	0	9 (60)
Academic degree	B.Sc.	112 (81.2)	0	0
	M.Sc.	26 (18.8)	0	0
	General practitioner	0	28 (100)	0
	Subspecialist	0	0	15 (100)
Major	Nursing	138 (100)	0	0
	Medicine	0	28 (100)	15 (100)

The results of the first phase of the study showed that all suggested educational topics were found necessary by the participants. According to the results, among the items included in the “main concepts and definition” section, “oxygen administration methods” (95.68%) and “risks and complications of oxygen administration” (92.26%) had the highest mean values. In the “oxygen therapy methods” section, “nasal cannula” (95.95%) and “trans-tracheal catheter” (90.51%) had the highest mean values. Table 2 shows the frequency distribution of the participants’ responses regarding the topics required in training the oxygen therapy methods.

**Table 2:** Participants’ responses about the topics required in training the oxygen therapy methods

NO	Oxygen therapy methods	Participants	Necessary		Mean Values	
			Fr (%)	Fr (%)	Necessary	Unnecessary
1	Nasal cannula	Nurses	136 (98.55)	2 (1.45)	**95.95**	4.05
		GPs	25 (89.28)	3 (10.71)		
		Specialists	15 (100)	0		
2	Simple mask	Nurses	96 (69.56)	42 (30.44)	**78.42**	21.58
		GPs	24 (85.71)	4 (14.29)		
		Specialists	12 (80)	3 (20)		
3	Re-breathing mask	Nurses	96 (69.56)	47 (34.06)	**78.24**	21.76
		GPs	24 (82.14)	4 (17.86)		
		Specialists	13 (86.66)	2 (13.34)		
4	Non-Rebreathing mask	Nurses	86 (62.31)	52 (37.69)	**59.81**	40.19
		GPs	16 (57.14)	12 (42.86)		
		Specialists	9 (60)	6 (40)		
5	Venture mask	Nurses	100 (72.46)	38(27.54)	**82.64**	17.36
		GPs	23 (82.14)	5 (17.86)		
		Specialists	14 (93.33)	1 (6.67)		
6	Trans-tracheal catheter	Nurses	127 (92.02)	11 (7.97)	**90.51**	9.49
		GPs	26 (92.85)	2 (7.14)		
		Specialists	13 (86.66)	2 (13.33)		
7	Aerosol Mask	Nurses	105 (76.08)	33 (23.91)	**75.83**	24.17
		GPs	20 (71.42)	8 (28.57)		
		Specialists	12 (80)	3 (20)		
8	T piece	Nurses	118 (85.50)	20 (14.50)	**81.35**	18.65
		GPs	22 (78.57)	6 (21.43)		
		Specialists	12 (80)	3 (20)		
9	Oxygen tent	Nurses	108 (78.26)	30 (21.72)	**66.33**	33.67
		GPs	17 (60.71)	11 (39.29)		
		Specialists	9 (60)	6 (40)		

### Developing an interactive e-learning course for oxygen therapy

**Fig. 1:**
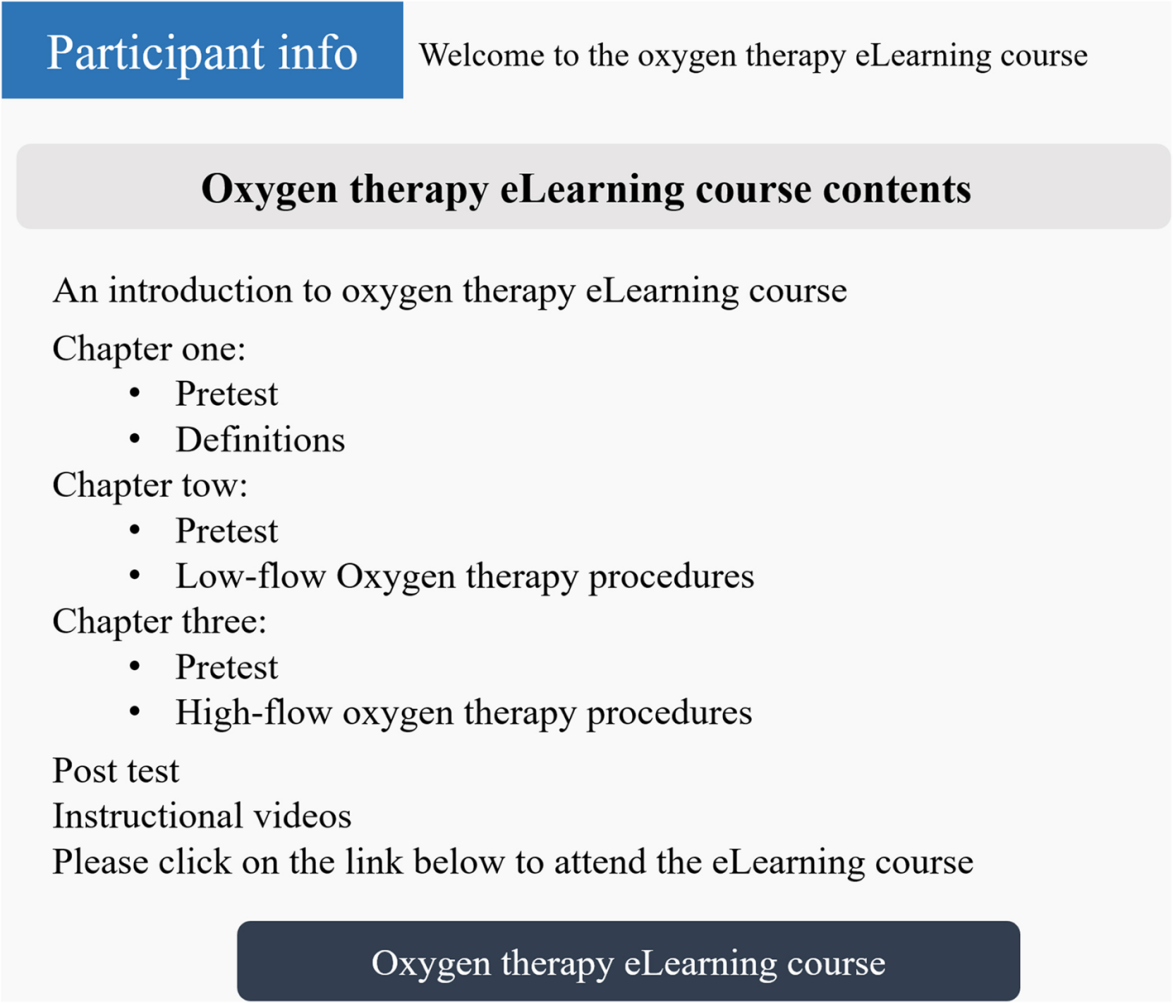
Course contents

**Fig. 2:**
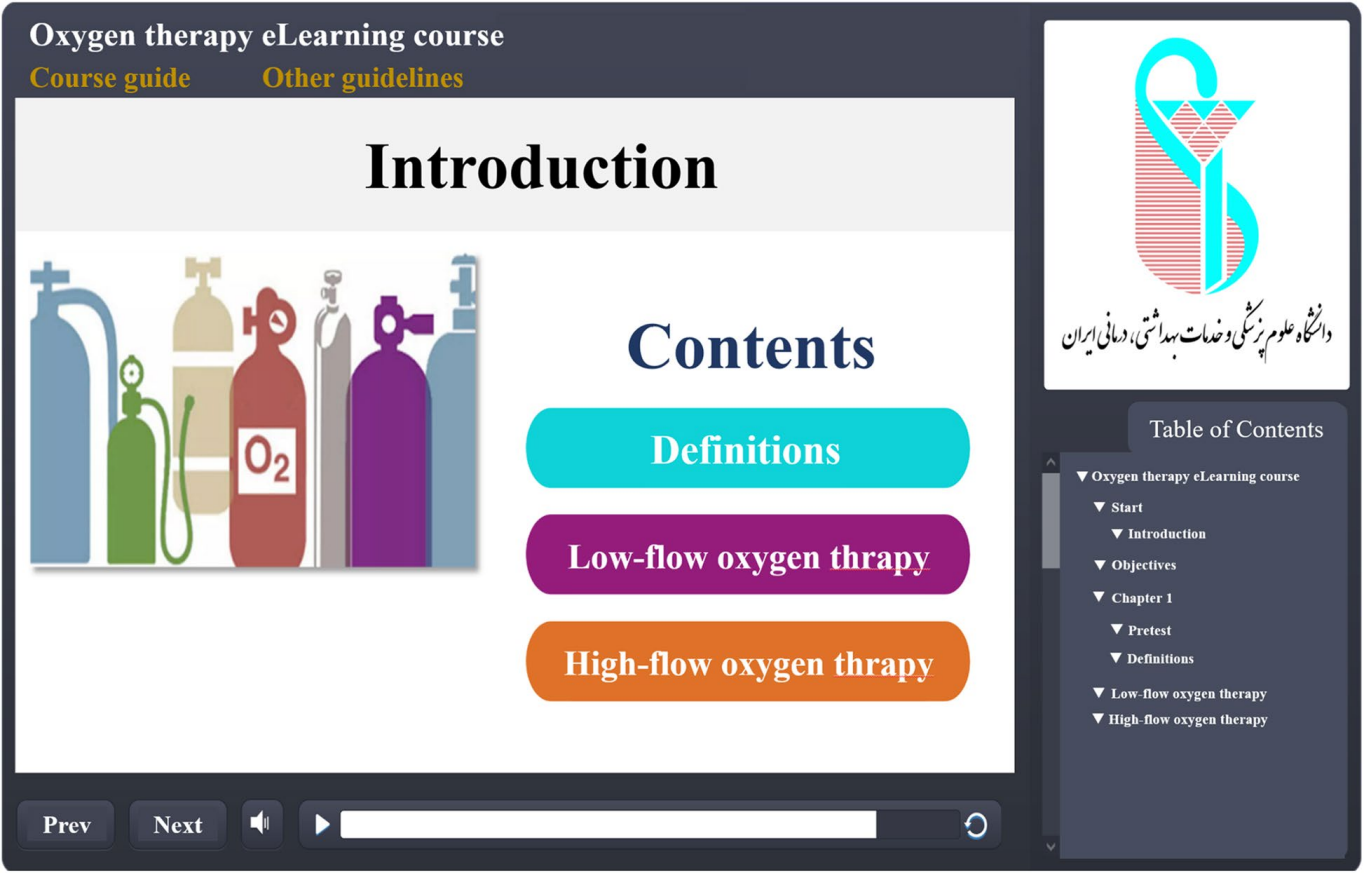
E-learning course overview

In the second phase of the research, based on the results derived from the first phase of the study, the required contents were extracted from the textbooks and reference books under the supervision of an emergency physician (HHB). Then, the prototype of the course was developed using interactive content production software, such as Camtasia, Flip PDF, and Story line software. The e-learning course had three main sections including “definitions”, “low pressure oxygen therapy methods”, and “high pressure oxygen therapy methods”. The contents of the course were voiced under the supervision of an emergency physician (HHB) and the pre-test and post-test questions were defined by him. Then, the contents were uploaded onto the website for easier access. The usernames and passwords were defined for the ED clinicians to be able to log into the website and access the training course. Figures 1 and 2 show some parts of the course contents.

### Evaluation of the e-learning course

#### Content evaluation

In the third phase of the research, 15 content production experts, who had at least 2 years of work experience in developing, producing, and evaluating e-learning courses, took part in the course evaluation process. The majority of the participants (*n* = 12, 80%) were female and about half of them (*n* = 8, 50%) belonged to the age group of 31 to 40. Table 3 shows the frequency distribution of the experts’ responses regarding the course evaluation.

**Table 3:** Experts’ opinions about the course contents

Criteria	Min mean value	Max mean value	Mean + SD
Cultural and literary criteria	4.66	4.86	4.66 + 0.33
Educational elements (objectives, content, methods, evaluation, and resources)	2.73	4.83	4.43 + 0.8
Course guide	3.46	4.66	3.91 + 1.07
Artistic and technological criteria	3.4	4.8	4.54 + 0.72

#### Comparisons between the pre- and post–test results

In the third phase of the research, 34 nurses, 5 general practitioners, and 7 emergency physicians passed the e-learning course. The highest frequency belonged to the age group of 31 to 40 (*n* = 18, 39.13%), and most of the participants were female (*n* = 30, 65.21%). In addition, the majority of the participants were nurses (*n* = 34, 73.91%). In order to evaluate the impact of the course on the participants’ knowledge, they were asked to complete the pre- and post-test questions. Then, their scores were compared, and the results showed that there was a statistically significant difference between the mean scores of pre-tests (10.61 + 2.42) and post-tests (14.47 + 2.1) of nurses (*p* < 0.001). The paired *t*-test for general practitioners also showed that there was a statistically significant difference (*p* < 0.002) between the mean scores of their pre-tests (15.4 + 0.48) and post-tests (18.3 + 1.3). Regarding the emergency physicians, there was no statistically significant difference (*p* > 0.05) between the mean scores of their pre-tests (18.42 + 0.53) and post-tests (19.28 + 075), although their mean scores increased in the post-test.

#### Usability evaluation

After completing the course, all participants were asked to complete a usability evaluation questionnaire to determine the usability level of the course, and 42 out of 46 clinicians (91.30%) participated. As Table 4 shows, on a scale of zero to 9, the “screen design” had the highest mean value (7.98 + 1.24), and “terminology and course information” (6.51 + 1.70) as well as general features (6.51 + 1.92) had the lowest mean values. As the mean values were higher than 6, it can be concluded that the participants evaluated the course at a good level.

**Table 4:** Usability evaluation of the course

Variables	Min mean value	Max mean value	Mean + SD
Overall reaction to the course	6.28	7.73	6.97 + 1.71
Screen design	7.23	8.71	7.98 + 1.24
Terminology and course information	5.9	7.4	6.51 + 1.70
Learnability	5.09	8	7.19 + 1.69
General features	3.92	7.66	6.51 + 1.92

## Discussion

Continuing education for health care professionals is important for improving their knowledge and quality of health care services. As a result, various online and in-person courses have been developed for different purposes and different groups of health care professionals [43, 44]. According to the literature, these courses can help clinicians to improve their performance, professional skills, confidence, and satisfaction [6, 14, 45–47].

The aim of this study was to provide an interactive e-learning course in oxygen therapy to improve the knowledge and skills of clinicians who worked in the emergency departments. In the first phase of this study, the educational needs of the ED clinicians were investigated by using a questionnaire. The findings showed that items, such as respiratory system function, physical assessment of the respiratory system, oxygen administration, non-invasive oxygen therapy, low flow oxygen administration, and high flow oxygen administration were considered necessary topics for training. Similarly, in a study conducted by Kho et al., non-invasive oxygen therapy methods such as simple mask, Venturi mask, re-breathing mask, non re-breathing mask; invasive oxygen therapy methods, such as invasive ventilation and endotracheal intubation as well as common medications for patients with respiratory problems were included in a blended learning course of emergency airway management [14]. In another study, to assess the efficacy of a standardized web-based training module for nurses in preparedness to fight against Covid-19, airway equipment, drug preparation, airway examination, and plans of airway management were demonstrated [48]. In Doshi et al.’s study, the learning scenarios were developed based on the face-to-face visits and treatment processes, and then, transformed to the e-learning modules [6].

In the present study, the anatomy of the respiratory system, non-invasive oxygen therapy methods, and some invasive oxygen therapy methods, such as intubation were considered as the main topics of the e-learning course. However, invasive oxygen therapy methods were not included, as they needed surgical skills.

To develop the e-learning course, different software, such as Storyline, Camtasia, and Flip PDF were used. The duration of the course depended on the time and speed of the participants, but in general it was developed as a 20-hour course. Clinicians could use the course intermittently or continuously depending on their time and job constraints. In order to provide the possibility of running the course on different platforms such as mobile phones, desktop computers, and tablets, this course was developed as a SCORM file, and uploaded onto the web-based platform. In Kho et al.’s study, part of the training course was presented in-person and practically, and the other part was presented electronically. The e-learning course included instructional videos and questions asked by the system, and participants could read the materials and answer the questions in the pre-defined time frame [14].

Finally, the e-learning course was evaluated. The evaluation study composed of three parts including content evaluation, impact evaluation and usability evaluation. The content production experts agreed that the course was developed in accordance with the recommended criteria for creating e-learning courses. In terms of impact evaluation, the results showed that there were statistically significant differences between the pre- and post-test scores of nurses and general practitioners. However, despite an increase in the mean scores of emergency physicians, no statistically significant difference was observed between their pre- and post-test scores.

Similarly, Najafi Ghezeljeh et al. used pre- and post-test questions to assess the nurses’ knowledge who participated in an e-learning course in delirium diagnosis. In this study, no statistically significant difference was found between the pre-test scores of the control and intervention groups. However, there was a statistically significant difference between the post-test scores of the intervention and control groups [49]. Samulski et al., also used pre- and post-test questions to measure the knowledge of the participants after passing a cytopathology online course. The results showed that there was a significant difference between their scores before and after participating in this course [50]. Similarly, Gupta et al. were successful in training nurses in airway management, as their scores significantly improved after passing the training program [48].

It seems that one of the important aspects in providing training courses, especially e-learning courses, is usability evaluation from the participants’ perspectives [51, 52]. In this study, the usability of the e-learning course was evaluated using the standard QUIS questionnaire. According to the results, the course participants evaluated the overall usability of the course at a good level. Similarly, Hersey and McAleer evaluated their course using a questionnaire which was designed based on the Kirkpatrick model. The results of this study showed that all nurses participated in the course were satisfied with the usability aspects [53]. In another study, Moreira et al. evaluated the usability of the course before the final test, and the results showed that all participants were completely satisfied with the training course [45]. Overall, it can be said that running an e-learning course in oxygen therapy was feasible and useful for ED clinicians. It can play an important role in improving their knowledge and professional skills, and can be used as a suitable alternative to the face-to-face continuing education courses.

### Research strengths and limitations

To the best of our knowledge, it was the first time in our country that such an e-learning course was developed for the ED clinicians. Investigating the educational requirements of the participants before designing the course was one of the main differences between the current study and other similar courses in which the contents were mainly developed based on the experts’ opinions or limited to the certain scenarios. Moreover, software design solutions were applied to make the course compatible with various devices, and different types of multimedia including text, audio, and video were used to make it more interactive. Evaluating the course from different aspects could be regarded as another strength of this study, in which content production experts’ opinions about maintaining the principles of e-learning course development, the ED clinicians’ knowledge before and after taking part in the course, and their perspectives about the course usability, which is a crucial activity in e-learning course development were investigated. In fact, we used a user-centered design approach to identify and address the real requirements of the ED clinicians both in selecting the contents of the course and designing the interface. The course also included contents related to oxygen therapy for patients with Covid-19, which can highlight its importance and usefulness, particularly for the ED clinicians during the Covid-19 pandemic.

However, the current study had some limitations. For example, data collection coincided with the Covid-19 pandemic and the number of the ED clinicians who participated in the course was limited. In addition, although this course could be used by different groups of health care providers, we focused on the ED clinicians, as they are in the front line of respiratory care services. Therefore, further studies are recommended to present the course to other clinicians, such as intensive care unit nurses. Adding other topics, such as invasive oxygen therapy and evaluating participants’ skills and performance can also increase the quality of the course. Another limitation of this research might be related to the small number of the emergency physicians who took part in the evaluation study. The small sample size might also affect the results of the study which showed that there was no statistically significant difference between the pre- and post-test scores of this group of the participants. Therefore, implementing the course in a larger sample size of emergency physicians are recommended to be able to compare the results.

## Conclusion

The purpose of this study was to develop and evaluate an e-learning course in oxygen therapy for the ED clinicians. To design the course, their educational requirements were investigated and the results showed that the course quality and usability were at the good level. Moreover, a statistically significant difference was observed between the pre- and post-test scores of nurses and general practitioners indicating that the course led to increasing their knowledge. Given the outbreak of Covid-19 and the importance of using oxygen therapy for patients, it is expected that passing this course would play an important role in improving the professional skills and knowledge of the participants. Furthermore, considering the working conditions of the ED clinicians, running e-learning courses seems to be more feasible than face-to-face courses. Therefore, this course can be used for continuing education of nurses and physicians in the EDs and other departments, such as intensive care units. Further investigations about the cost-effectiveness of the course and its impact on the clinicians’ knowledge and quality of patient care are also recommended.

## Data Availability

All data generated or analyzed during this study are included in this article.

## Abbreviations

ED: Emergency Department
ICT: Information and Communication Technology
ICU: Intensive Care Unit
NIH: National Institute of Health
WHO: World Health Organization
